# Amelioratory Effects of Testosterone Propionate on Age-related Renal Fibrosis via Suppression of TGF-β1/Smad Signaling and Activation of Nrf2-ARE Signaling

**DOI:** 10.1038/s41598-018-29023-3

**Published:** 2018-07-16

**Authors:** Guoliang Zhang, Yunxiao Kang, Chenming Zhou, Rui Cui, Min Jia, Shen Hu, Xiaoming Ji, Jiayu Yuan, Huixian Cui, Geming Shi

**Affiliations:** 1 Department of Neurobiology, Hebei Medical University, Shijiazhuang, 050017 PR China; 2 Department of Human Anatomy, Hebei Medical University, Shijiazhuang, 050017 PR China; 3 Department of electron microscopy center, Hebei Medical University, Shijiazhuang, 050017 PR China; 4 Department of urology, Hebei Civil Affairs General Hospital, Xingtai, 054000 PR China; 5 Department of urology, Handan Second hospital, Handan, 056001 PR China; 6 Neuroscience Research Center, Hebei Medical University, Shijiazhuang, 050017 PR China

## Abstract

Androgen plays a pivotal role in the progression of renal fibrosis. However, whether exogenous androgen treatment to aged male rats can improve the age-related renal fibrosis was not explored. In our study, the changes of morphological structure, renal fibrosis, ultrastructure and renal function, the expressions of extracellular matrix (ECM), matrix metalloproteinases (MMPs) and its tissue inhibitors of metalloproteinases (TIMPs), the expressions of tumor growth factor β1 (TGF-β1)/Smad signaling and oxidative stress parameters as well as nuclear factor erythroid 2-related factor 2-antioxidant response element (Nrf2-ARE) signaling were tested in kidney of aged male Wistar rats after subcutaneous testosterone propionate (TP, 2 mg/kg/d, 84-day) injection. Aged rats showed significantly renal histopathological changes, increased renal fibrosis, increased thickening of the glomerular basement membrane and the Bowman’s capsule basement membrane, declined renal functional, increased ECM, lower expressions of MMP-2 and MMP-9 and higher expressions of TIMP-1 and TIMP-2 in renal tissues and higher expressions of TGF-β1/Smad signaling, as well as lower expressions of Nrf2-ARE signaling compared to young rats. TP treatment significantly improved age-related above indexes. These results suggested that TP supplement may alleviate age-related renal fibrosis via suppression of TGF-β1/Smad signaling and activation of Nrf2-ARE signaling in aged rats.

## Introduction

Aged kidney was commonly accompanied with structural and physiologic changes^1,2^. Renal fibrosis was a major damage of age-related progressive kidney disease^3,4^. The cellular mechanisms that lead to age-related renal fibrosis were complex including inflammation, oxidative stress, apoptosis and senescence^5^. Therefore, the efficient therapeutic strategies were great significance in the control of age-related renal fibrosis. Renal fibrosis is characterized by the accumulation of extracellular matrix (ECM) proteins^6,7^. Matrix metalloproteinases (MMPs) were multifunctional enzymes capable of cleaving the basal membrane and ECM components. MMPs activity is regulated via a number of mechanisms, including inhibition by tissue inhibitors of metalloproteinases (TIMPs)^8,9^.

Increasing evidence shows that tumor growth factor β (TGF-β) signaling is known to play a key role in the renal fibrosis^10,11^. TGF-β1 was considered as a pivotal mediator in renal fibrosis by activating its downstream Smad signaling pathway^11,12^. TGF-β1 initiates renal fibrosis, whereas MMPs, TIMPs and ECM may act in further stages of this process. In addition, oxidative stress was one of the mechanisms participated in the age-related renal fibrosis. Reactive oxygen species (ROS) play an important role as high levels of oxidative stress. ROS including hydrogen peroxide (H_2_O_2_), lipid peroxides (LPO), superoxide anions and hydroxyl radicals generated during normal cellular oxidative metabolism. Malondialdehyde (MDA) and LPO were lipid peroxidation parameters^13^. The activities of several antioxidant enzymes including glutathione catalase (CAT), superoxide dismutase (SOD) and peroxidase (GSH-px), as well as non enzymatic antioxidants glutathione (GSH) levels resulted in ROS production^14^. The nuclear factor erythroid 2-related factor 2-antioxidant response element (Nrf2-ARE) pathway regulates cellular responses to oxidative and electrophilic stress. In the aging process, the circulating level of testosterone was progressive reduction in males^15,16^. Androgen was capable of modulating two important cellular components, namely ECM accumulation and oxidative stress, however, its anti-renal fibrosis characters were not explored. The efficacy of testosterone propionate (TP) replacement was controversial in animal experiments^17^. The different organisms studied and the treatment regimen of androgens was important factors. Based on the effects of oxidative stress on aging-related kidney fibrosis, the status of oxidative stress in organisms might be the candidate for the discrepancy when androgens were supplemented.

In the present studies, the changes of kidney morphology and function in aged rats were observed after TP administration as well as the blocking effects in renal fibrosis of TP administration was examined by exploring the TGF-β1/Smad and the Nrf2-ARE signaling pathway.

## Results

Concentration of serum testosterone was lower in 24Mon rats (1.58 ± 0.24 ng/ml) compared to 6Mon rats (4.08 ± 0.55 ng/ml, *P* < 0.01). Supplement of TP increased the concentration of testosterone in 24Mon-TP rats (12.59 ± 1.91 ng/ml) compared to 24Mon rats (*P* < 0.01).

### The effects of TP on renal morphology in aged rats

**Figure 1:**
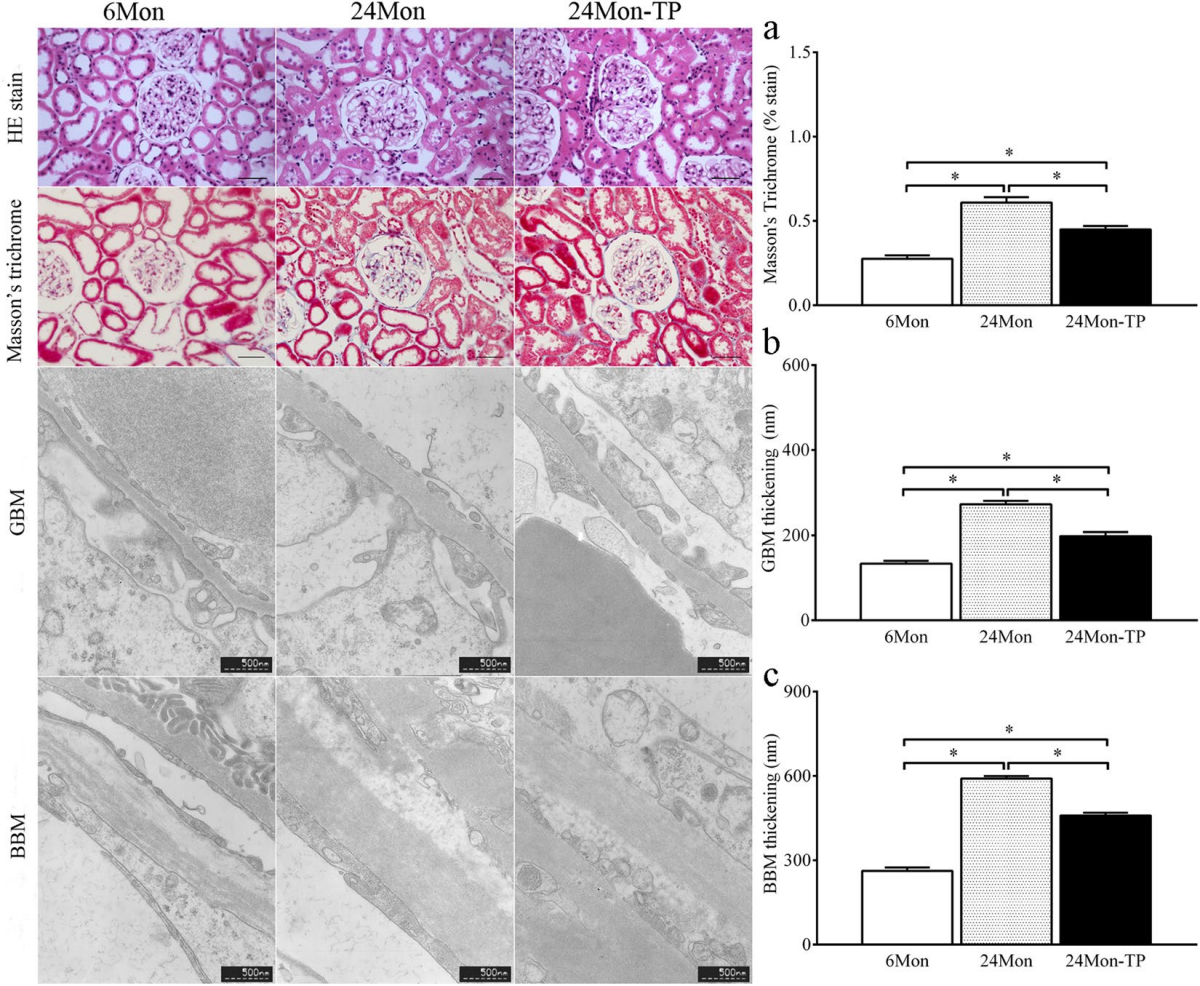
H&E stain (Bar = 50 μm), Masson’s trichrome stain and transmission electron microscope (TEM) of renal tissues. Renal interstitial fibrosis was assessed from Masson’s trichrome stained. (**a**) Average percentage positive stain of Masson’s trichrome (blue). Bar = 50 μm. Changes in the structure of the glomeruli were observed by transmission electron microscope. (**b**) GBM thickening. Bar = 500 nm. (**c**) BBM thickening. Bar = 500 nm.

The 6Mon group showed regular morphology with no evidence of histopathological changes. The 24Mon group showed prominent degenerative changes with tubular degeneration, tubular cell swelling and cellular vacuolization as well as glomerular degeneration in the renal cortical tissues. On the contrary, TP supplement was able to restore age-related renal histopathological changes (Fig. 1).

### The effects of TP on renal fibrosis in aged rats

Compared to 6Mon rats, the average percentage positive stain in 24Mon rats was increased significantly (*P* < 0.01). Average percentage positive stain of 24Mon-TP rats was decreased by 26.3% after TP treatment than that of 24Mon rats (*P* < 0.01) and was not restored to the level of 6Mon rats (*P* < 0.01) (Fig. 1a).

### The effects of TP on the thickening of glomerular basement membrane (GBM) and Bowman’s capsule basement membrane (BBM) of aged rats

Compared to 6Mon rats, the thickening of GBM and BBM in 24Mon rats was increased significantly (*P* < 0.01). The thickening of GBM and BBM of 24Mon-TP rats was decreased by 27.3% and 22.3% after TP treatment than that of 24Mon rats (*P* < 0.01) and was not restored to the level of 6Mon rats (*P* < 0.01) (Fig. 1).

### The effects of TP on renal function in aged rats

**Figure 2:**
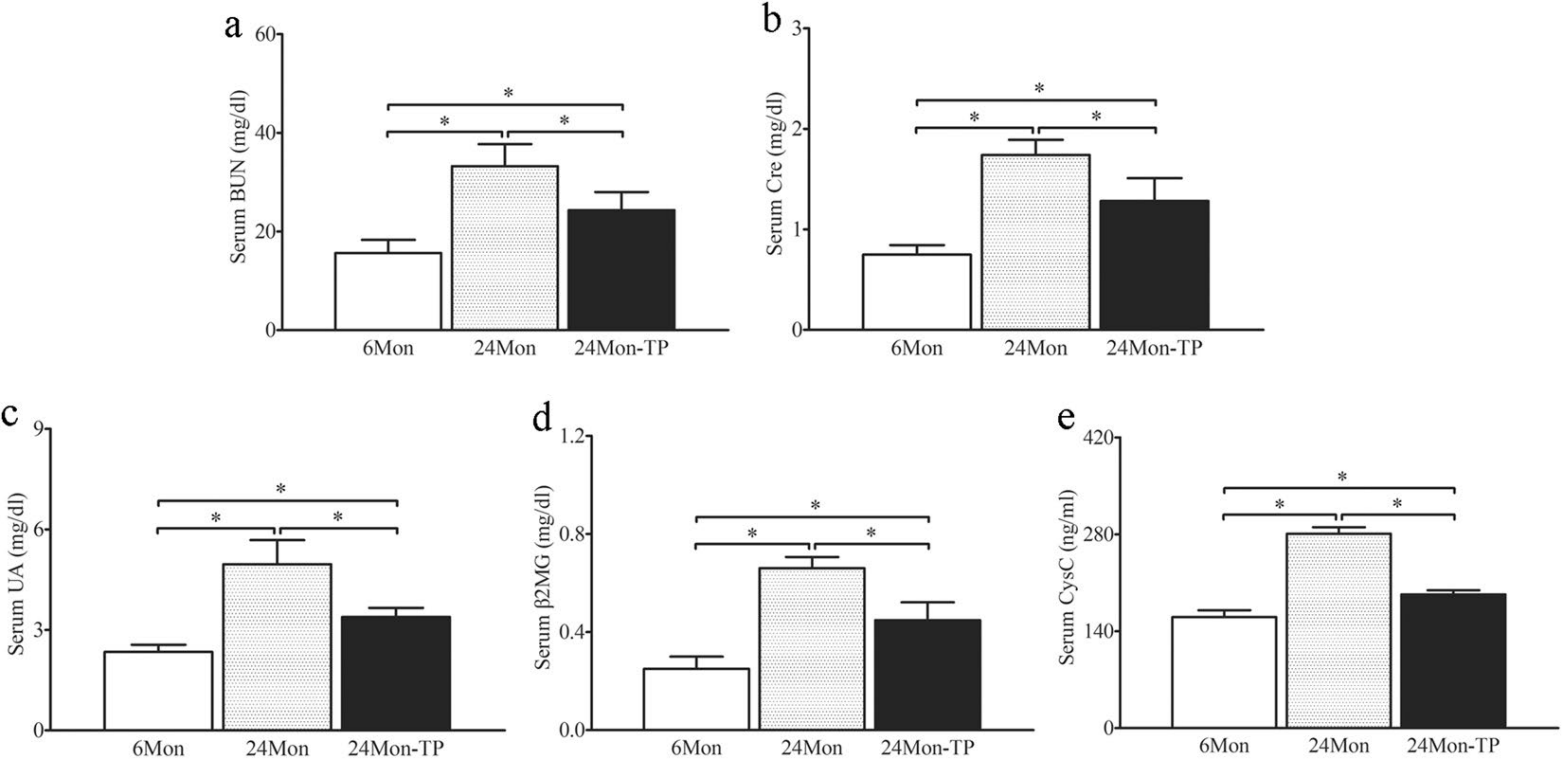
The effects of TP on renal function in aged rats. The serum level of BUN (**a**), Cre (**b**), UA (**c**), β2MG (**d**) and CysC (**e**) was measured by ELISA. The asterisks show significant differences (*P < 0.01).

Compared to 6Mon rats, the serum level of blood urea nitrogen (BUN, Fig. 2a), Creatinine (Cre, Fig. 2b), Uric acid (UA, Fig. 2c), β2-microglobulin (β2MG, Fig. 2d) and Cystatin C (CysC, Fig. 2e) in 24Mon rats was increased significantly (*P* < 0.01). The level of BUN, Cre, UA, β2MG and CysC of 24Mon-TP rats were decreased by 26.8%, 26.3%, 31.7%, 32.1% and 31.2% respectively after TP treatment than that of 24Mon rats (*P* < 0.01). The serum level of BUN, Cre, UA, β2MG and CysC of 24Mon-TP rats were not restored to the level of 6Mon rats (*P* < 0.01) (Fig. 2).

### The effects of TP on expression of Collagen I, Collagen IV and fibronectin in the renal tissue of aged rats

**Figure 3:**
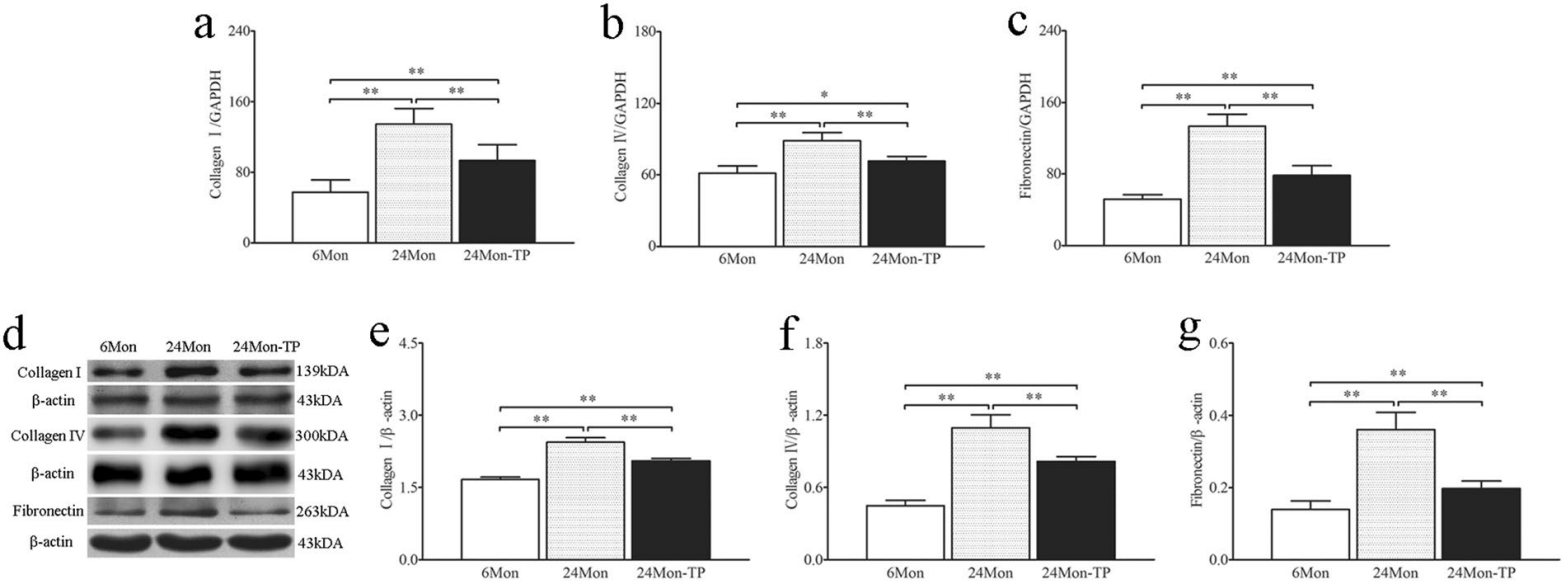
The effects of TP on the Collagen I, Collagen IV and fibronectin in the renal tissue of aged rats. Bar graphs show Collagen I mRNA (**a**), Collagen IV mRNA (**b**) and fibronectin mRNA (**c**). (**d**) Western blot study revealed Collagen I, Collagen IV and fibronectin expression in the renal tissue. Bar graphs illustrate the protein expression of Collagen I (**e**) Collagen IV (**f**) and fibronectin (**g**). The asterisks show significant differences (*P < 0.05, **P < 0.01).

Compared to 6Mon rats, the expression of Collagen I mRNA (Fig. 3a), Collagen IV mRNA (Fig. 3b) and fibronectin mRNA (Fig. 3c) in 24Mon rats was increased significantly (*P* < 0.01). The expression of Collagen I mRNA, Collagen IV mRNA and fibronectin mRNA of 24Mon-TP rats was lower by 30.5%, 19.6% and 41.1% respectively than that of 24Mon rats (*P* < 0.01) and was not restored to the level of 6Mon rats (*P* < 0.05) (Fig. 3a–c). By Western blot, Collagen I, Collagen IV and fibronectin were located at ~139 kDa, ~300 kDa and ~263 kDa respectively (Fig. 3d). Compared to 6Mon rats, the expression of Collagen I (Fig. 3e), Collagen IV (Fig. 3f) and fibronectin (Fig. 3g) protein in 24Mon rats was increased significantly (*P* < 0.01). The expression of Collagen I, Collagen IV protein and fibronectin of 24Mon-TP rats was lower by 15.9%, 25.6% and 45.4% respectively than that of 24Mon rats (*P* < 0.01) and was not restored to the level of 6Mon rats (*P* < 0.01) (Fig. 3e–g).

### The effects of TP on expression of MMP-2, MMP-9, TIMP-1 and TIMP-2 in the renal tissue of aged rats

**Figure 4:**
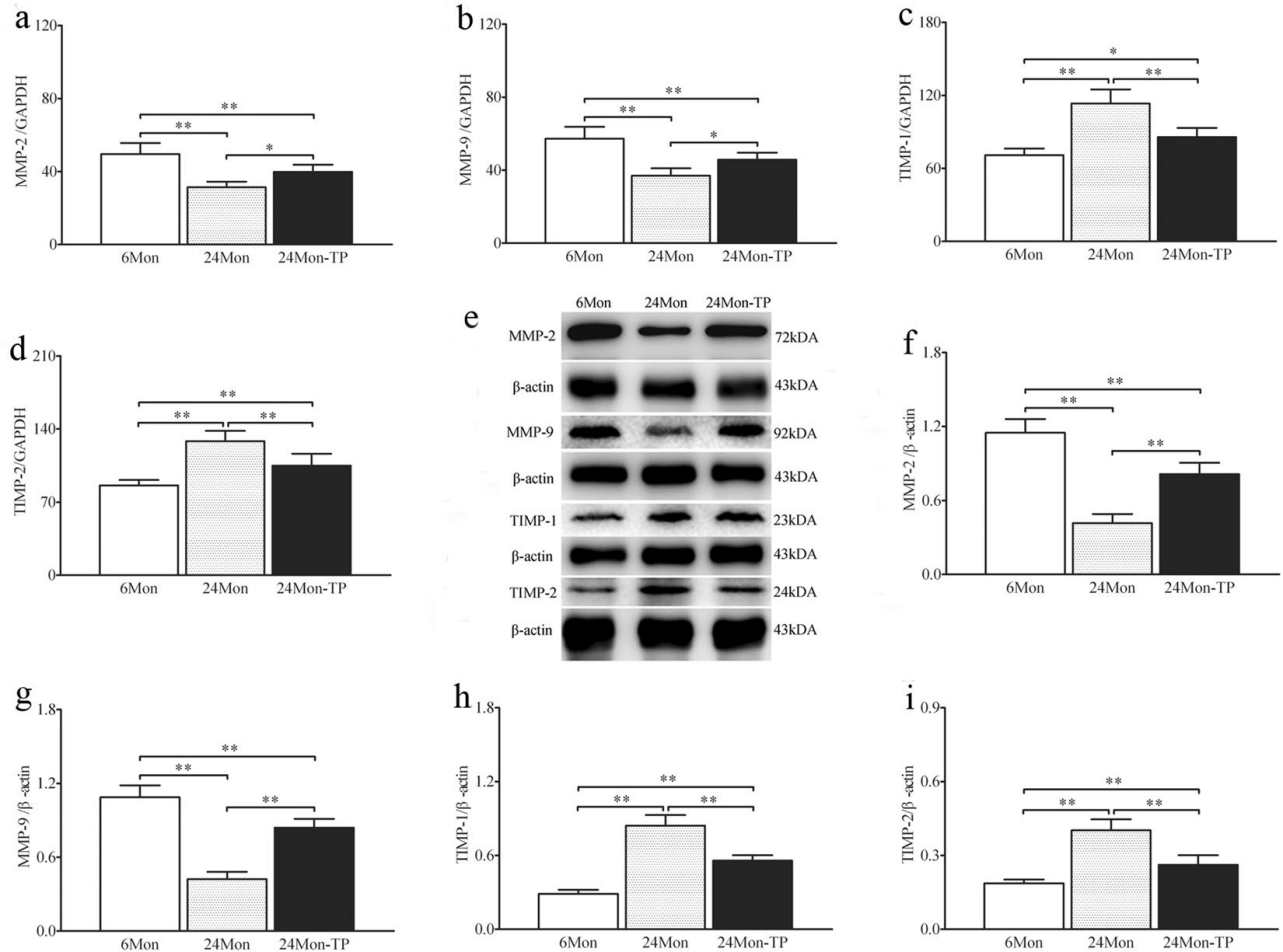
The effects of TP on the MMP-2, MMP-9, TIMP-1 and TIMP-2 in the renal tissue of aged rats. Bar graphs show MMP-2 mRNA (**a**), MMP-9 mRNA (**b**), TIMP-1 mRNA (**c**) and TIMP-2 mRNA (**d**). (**e**) Western blot study revealed MMP-2, MMP-9, TIMP-1 and TIMP-2 expression in the renal tissue. Bar graphs illustrate the protein expression of MMP-2 (**f**), MMP-9 (**g**), TIMP-1 (**h**) and TIMP-2 (**i**). The asterisks show significant differences (*P < 0.05, **P < 0.01).

Compared to 6Mon rats, the expression of MMP-2 mRNA (Fig. 4a) and MMP-9 mRNA (Fig. 4b) in 24Mon rats was decreased significantly (*P* < 0.01) and the expression of TIMP-1 mRNA (Fig. 4c) and TIMP-2 mRNA (Fig. 4d) in 24Mon rats was increased significantly (*P* < 0.01). The expression of MMP-2 mRNA and MMP-9 mRNA of 24Mon-TP rats were increased by 26.9% and 23.6% respectively (*P* < 0.05) and the expression of TIMP-1 mRNA and TIMP-2 mRNA of 24Mon-TP rats lower by 24.4% and 18.4% respectively (*P* < 0.01) than that of 24Mon rats. The expression of MMP-2 mRNA (*P* < 0.01), MMP-9 mRNA (*P* < 0.01), TIMP-1 mRNA (*P* < 0.05) and TIMP-2 mRNA (*P* < 0.01) of 24Mon-TP rats was not restored to the level of 6Mon rats (Fig. 4a–d). By Western blot, MMP-2, MMP-9, TIMP-1 and TIMP-2 were located at ~72 kDa, ~92 kDa, ~23 kDa and ~24 kDa respectively (Fig. 4e). Compared to 6Mon rats, the expression of MMP-2 (Fig. 4f) and MMP-9 (Fig. 4g) in 24Mon rats was significantly decreased and the expression of TIMP-1 (Fig. 4h) and TIMP-2 (Fig. 4i) in 24Mon rats was increased significantly (*P* < 0.01). The expression of MMP-2 and MMP-9 of 24Mon-TP rats were increased by 94.7% and 98.9% respectively and the expression of TIMP-1 and TIMP-2 of 24Mon-TP rats lower by 33.8% and 34.8% respectively than that of 24Mon rats(*P* < 0.01). The expression of MMP-2, MMP-9, TIMP-1 and TIMP-2 of 24Mon-TP rats was not restored to the level of 6Mon rats (*P* < 0.01) (Fig. 4f–i).

### The effects of TP on expression of TGF-β1/Smad signaling in the renal tissue of aged rats

**Figure 5:**
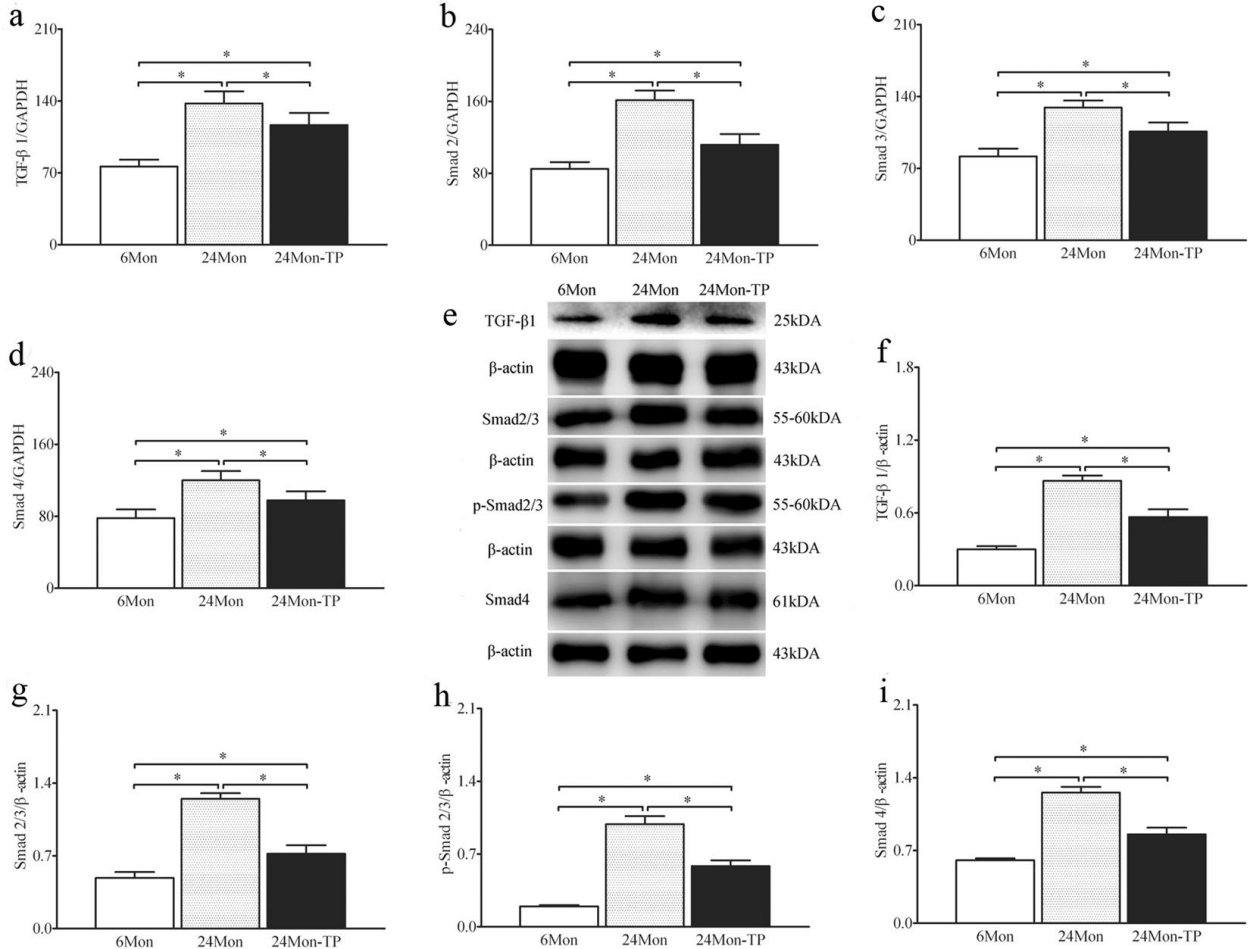
The effects of TP on the TGF-β1/Smad signaling in the renal tissue of aged rats. Bar graphs show TGF-β1 mRNA (**a**), Smad 2 mRNA (**b**), Smad 3 mRNA (**c**) and Smad 4 mRNA (**d**). (**e**) Western blot study revealed TGF-β1, Smad 2/3, p-Smad 2/3 and Smad 4 expression in the renal tissue. Bar graphs illustrate the protein expression of TGF-β1 (**f**), Smad 2/3 (**g**), p-Smad 2/3 (**h**) and Smad 4 (**i**). The asterisks show significant differences (*P < 0.01).

Compared to 6Mon rats, the expression of TGF-β1 mRNA (Fig. 5a), Smad 2 mRNA (Fig. 5b), Smad 3 mRNA (Fig. 5c) and Smad 4 mRNA (Fig. 5d) in 24Mon rats was increased significantly (*P* < 0.01). The expression of TGF-β1 mRNA, Smad 2 mRNA, Smad 3 mRNA and Smad 4 mRNA of 24Mon-TP rats lower by 15.2%, 30.7%, 18.1% and 18.7% respectively than that of 24Mon rats (*P* < 0.01) and was not restored to the level of 6Mon rats (*P* < 0.01) (Fig. 5a–d). By Western blot, TGF-β1, Smad 2/3, phosphorylated-Smad 2/3 (p-Smad 2/3) and Smad 4 protein were located at ~25 kDa, ~55–60 kDa, ~55–60 kDa and ~61 kDa respectively (Fig. 5e). Compared to 6Mon rats, the expression of TGF-β1 (Fig. 5f), Smad 2/3 (Fig. 5g), p-Smad 2/3 (Fig. 5h) and Smad 4 (Fig. 5i) in 24Mon rats was increased significantly (*P* < 0.01). The expression of TGF-β1, Smad 2/3, p-Smad 2/3 and Smad 4 of 24Mon-TP rats lower by 34.6%, 42.3%, 40.8% and 32.0% respectively than that of 24Mon rats (*P* < 0.01) and was not restored to the level of 6Mon rats (*P* < 0.01) (Fig. 5f–i).

### The effects of TP on oxidative stress parameters in the renal tissue of aged rats

**Figure 6:**
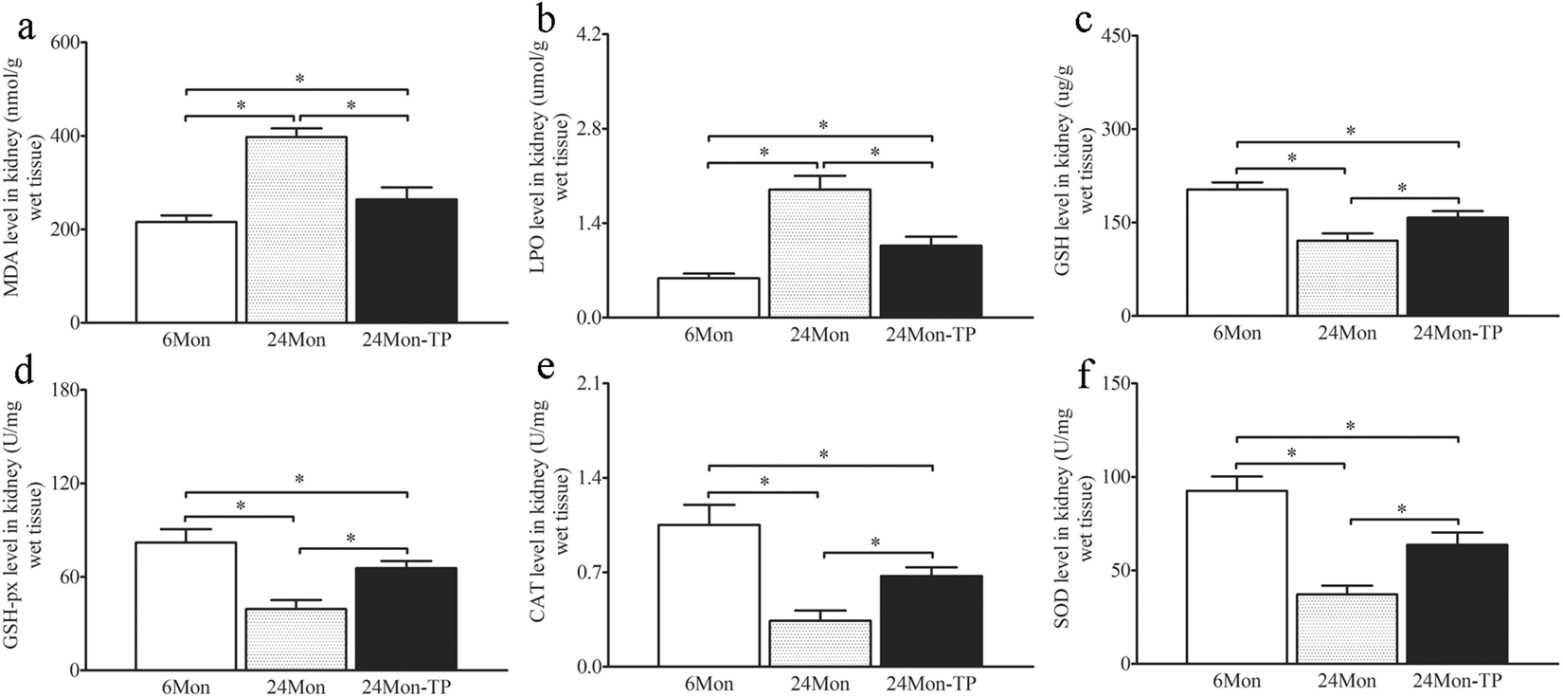
The effects of TP on oxidative stress parameters in the renal tissue of aged rats. MDA (**a**), LPO (**b**), GSH (**c**), GSH-px (**d**), CAT (**e**) and SOD (**f**) levels of the renal tissue were measured. The asterisks show significant differences (*P < 0.01).

Compared to 6Mon rats, the levels of MDA (Fig. 6a) and LPO (Fig. 6b) of 24Mon rats were significantly increased (*P* < 0.01) and the levels of GSH (Fig. 6c), GSH-px (Fig. 6d), CAT (Fig. 6e) and SOD (Fig. 6f) of 24Mon rats were reduced significantly (*P* < 0.01). The levels of MDA and LPO of 24Mon-TP rats were decreased by 33.5% and 43.9% respectively and the levels of GSH, GSH-px, CAT and SOD of 24Mon-TP rats were increased by 23.5%, 40.0%, 49.4% and 41.6% respectively than that of 24Mon rats after TP administration (*P* < 0.01) and were not restored to the level of 6Mon rats (*P* < 0.01) (Fig. 6).

### The effects of TP on Nrf2-ARE signaling in the renal tissue of aged rats

**Figure 7:**
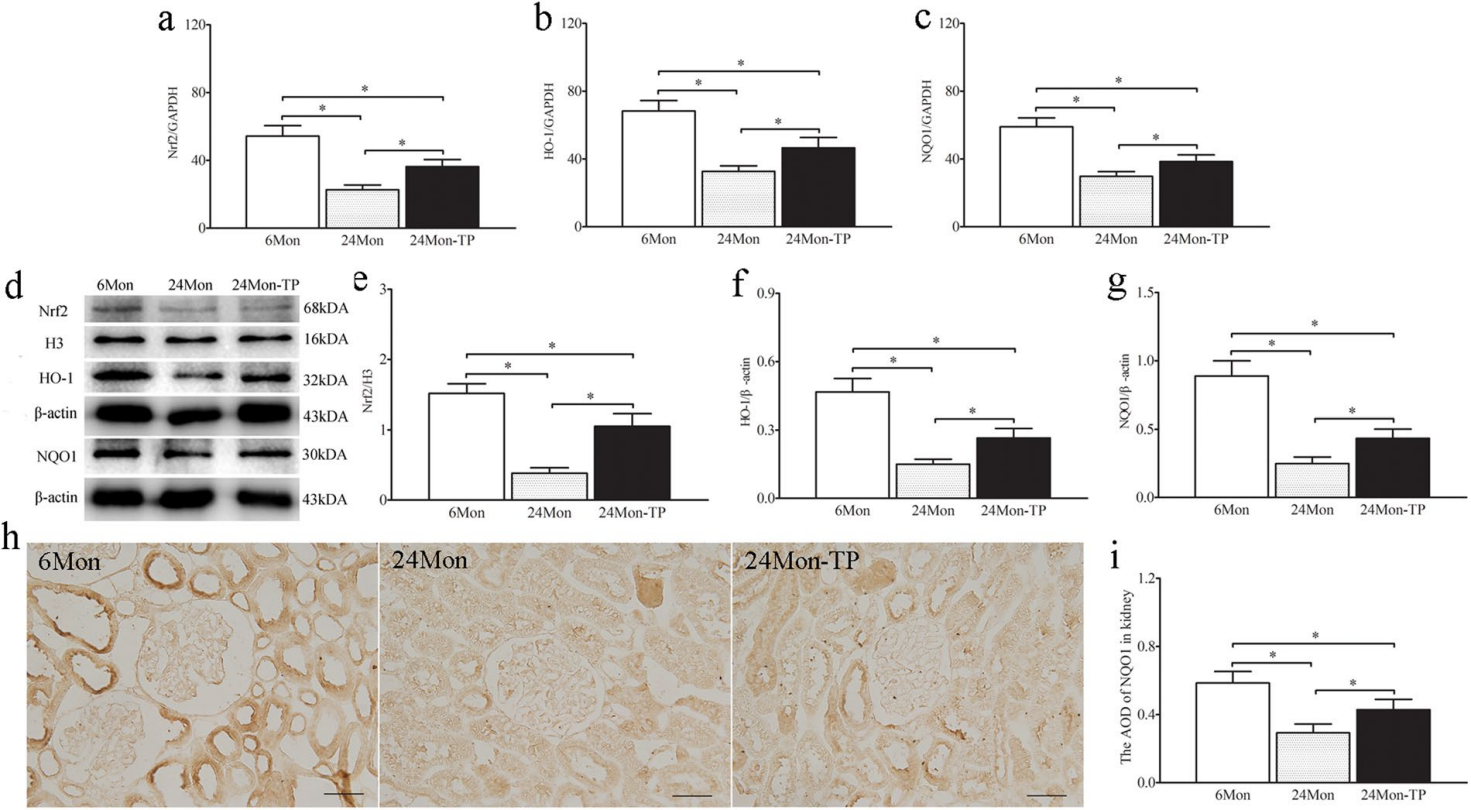
The effects of TP on Nrf2-ARE signaling in the renal tissue of aged rats. Bar graphs showed Nrf2 mRNA (**a**), HO-1 mRNA (**b**) and NQO1 mRNA (**c**). (**d**) Western blot study revealed Nrf2, HO-1 and NQO1 expression in the renal tissue. Bar graphs illustrated the protein expression of Nrf2 (**e**), HO-1 (**f**) and NQO1 (**g**). (**h**) Immunohistochemistry revealed the variation of NQO1 immunoreactive intensity. Bar = 500 nm. (**i**) Bar graph showed the AOD of NQO1. The asterisks show significant differences (*P < 0.01).

Compared to 6Mon rats, the expression of Nrf2 mRNA (Fig. 7a), HO-1 mRNA (Fig. 7b) and NQO1 mRNA (Fig. 7c) in 24Mon rats was decreased significantly (*P* < 0.01). The expression of Nrf2, HO-1 and NQO1 mRNA of 24Mon-TP rats increased by 60.3%, 42.6% and 29.3% respectively than that of 24Mon rats (*P* < 0.01) and was not restored to the level of 6Mon rats (*P* < 0.01) (Fig. 7a–c). By Western blot, Nrf2, HO-1 and NQO1 protein were located at ~68 kDa, ~32 kDa and ~30 kDa respectively (Fig. 7d). Compared to 6Mon rats, the expression of Nrf2 (Fig. 7e), HO-1 (Fig. 7f) and NQO1 (Fig. 7g) in 24Mon rats was decreased significantly (*P* < 0.01). The expression of Nrf2, HO-1 and NQO1 of 24Mon-TP rats increased by 175.6%, 76.9% and 74.8% respectively than that of 24Mon rats (*P* < 0.01) and was not restored to the level of 6Mon rats (*P* < 0.01) (Fig. 7d–g). By immunohistochemistry revealed the variation of NQO1 immunoreactive intensity (Fig. 7h). Compared to 6Mon rats, the AOD of NQO1 (Fig. 7i) in 24Mon rats was decreased significantly (*P* < 0.01). Compared to 24Mon rats, the NQO1 of 24Mon-TP rats increased by 46.5% (*P* < 0.01) and was not restored to the level of 6Mon rats (*P* < 0.01) (Fig. 7i).

## Discussion

**Figure 8:**
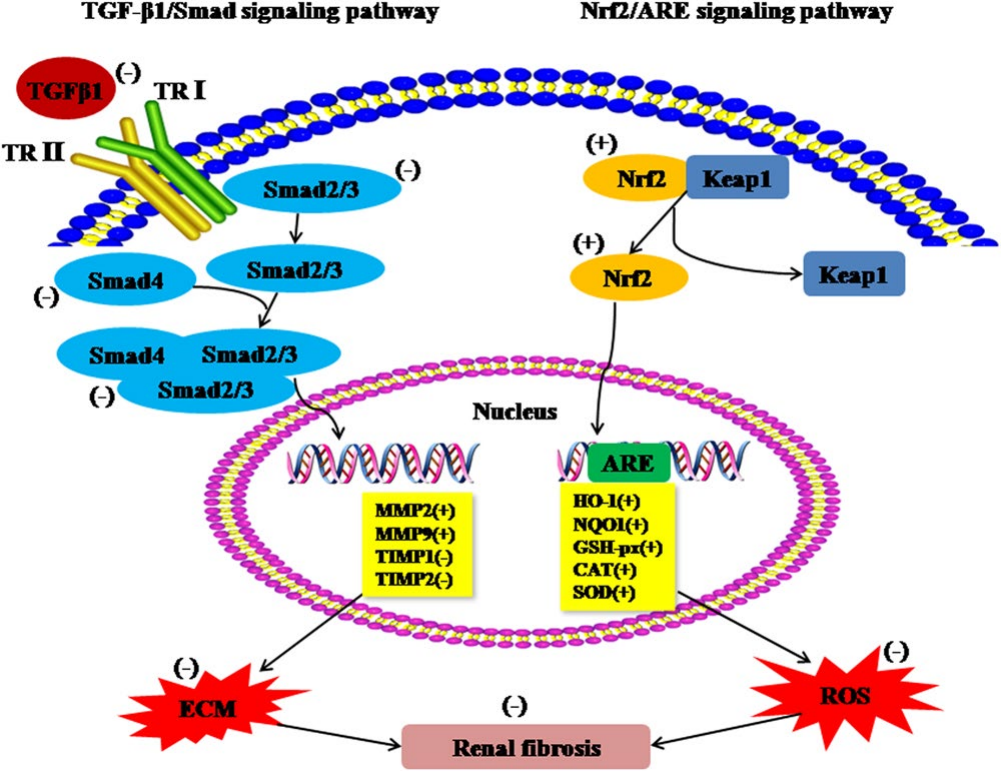
Regulatory effects of androgen on TGF-β1/Smad signaling pathway and Nrf2-ARE signaling pathway. (+): positive effect; (−): negative effect.

In the present studies, the results showed that TP tretment ameliorated the age-related renal histopathological change, renal function, ECM, MMPs, TIMPs and TGF-β1/Smad signaling as well as oxidative stress and Nrf2-ARE signaling of the aged rats. Structural changes and declined in renal function were significantly improved in aged rats following TP injection. The expression of Collagen I, Collagen IV and fibronectin, as the important factor of ECM, significantly decreased after chronic TP treatment. The increased expression of MMP-2 and MMP-9 and decreased expression levels of TIMP-1 and TIMP-2 in renal tissues, the inhibition of TGF-β1/Smad signaling, as well as the activation of Nrf2-ARE signaling were found after chronic TP supplement. The results indicated that chronic supplementation of TP may act as an antifibrotic agent via suppression of TGF-β1/Smad signaling and activation of Nrf2-ARE signaling (Fig. 8).

In the pathogenesis of renal senescence, several structural changes occur with aging which including glomerulosclerosis, thickening of the basement membrane, tubulointerstitial fibrosis, decreased in tubular number, volume and length^18^. There was a linear relationship between aging and a decline in renal function. The expression of collagen IV was showed age-related increases in 24 and 30-month-old rats^5^. It is well known that ECM accumulation is the ultimately pathway to cause renal fibrosis with aging^3,19^. Imbalance of MMPs/TIMPs results in age-related renal fibrosis^20,21^. These findings were consistent with our studies. The mechanisms involved in the development of the morphological and functional changes associated with aging have not been exactly elucidated. In present studies, TP replacement therapy improved kidney structure, decreased the thickening of GBM and BBM and renal function as well as decreased fibrosis level (Masson’s trichrome staine) and ECM deposition in aged rats. Therefore, we presumed that TP may be regulating the MMPs and TIMPs.

In previous studies showed that ECM degradation is catalyzed by MMPs, which including collagenases, gelatinases, stromelysins, matrilysins as well as membrane-type MMPs^22,23^. In particular, MMP-2 and MMP-9 were associated with the renal fibrosis^24^. The activities of MMPs are inhibited by TIMPs. In TIMPs family, TIMP-1 and TIMP-2 were capable of inhibiting the activities of MMPs, and play a important role in maintaining the balance between ECM deposition^25^. With down-regulation of MMPs and up-regulation of TIMPs in renal fibrosis of the rabbit model of unilateral ureteral obstruction, collagen IV degradation was inhibited, which promoted ECM accumulation^26^. In our results, we found that the activity of MMP-2 and MMP-9 was declined and the activity of TIMP-1 and TIMP-2 was increased in aged rats. Chronic TP supplementation increases the expression of MMP-2 and MMP-9 as well as decreases the expression of TIMP-1 and TIMP-2. These data demonstrated that ECM accumulation was always accompanied by the changes of MMPs and TIMPs during age-related renal fibrosis, TP can regulate the balance between MMPs and TIMPs.

Increasing evidence shows that TGF-β1/Smad signaling was a central pathway leading to tissue fibrosis^27^. TGF-β1 exerts its effects via the TGF-β1 signaling cascade occurs upon TGF-β1 binding to TGF-β type II receptor (TRII). TRII can transphosphorylates the type I receptor (TRI) and subsequent TRI-TRII hetero-tetrameric complex formation, resulting in phosphorylation of Smad 2/3. Subsequently, p-Smad 2/3 binds to the common Smad 4 and form the Smad complex to translocate into the nucleus to regulate the target gene transcription^10,11,28^. Testosterone attenuated Smad 2/3 phosphorylation mediated by TGF-β1, which can result in decreased cardiac fibroblast activation and potentially contribute to beneficial effects in heart failure^29,30^. The androgen possesses protective effects against angiotensin II-induced vascular remodeling by regulating the TGF-β1/Smad signaling. Our results showed that aged rats received chronic TP treatment maintained the lower expression of TGF-β1, Smad 2/3, p-Smad 2/3 and Smad 4, which suggested that TP plays a role in negatively regulating TGF-β1/Smad signaling.

The renal fibrosis-associated oxidative stress is due to increased ROS and diminished antioxidant capacity^31,32^. Mounting evidence has demonstrated that oxidative stress is involved in increased lipid peroxidation^33,34^. Nrf2 was a key transcription factor in defending against oxidative stress through modulating its downstream antioxidant and detoxifying enzymes^35^. Activation of the Nrf2-ARE signaling ameliorated organ fibrosis^28,36,37^. Nrf2 is normally distributed throughout the cytoplasm associated with Kelch-like ECH associated protein 1 (Keap-1)^38,39^. Under conditions of oxidative stress, the activity of Keap1 is diminished and Nrf2 evades Keap1-mediated degradation, translocates to the nucleus where it activates the high expression of its target genes^40^. In the present study, TP treatment significantly decreased the levels of MDA and LPO, and increased the activity of antioxidant enzymes, GSH, GSH-px, CAT and SOD, increased the expression of Nrf2, HO-1 and NQO1 in renal tissue of aged rats. Therefore, the protective effects of TP in renal fibrosis may partly depend on activation of the Nrf2-ARE signaling and then decreased the ROS. To our knowledge, the results from our study represent the first evidence showing that TP protects against renal fibrosis through the activation of Nrf2-ARE signaling.

Testosterone plays its role in multiple physiological functions which including growth, development, secretory function and survival^41,42^. Testosterone act on target cells by activating specific nuclear receptors which regulate the transcription of a variety of genes^43,44^. Many reports demonstrated that testosterone have been associated with a variety of adverse effects that impact many organ systems. Testosterone replacement was beneficial in young androgen-deficient men with an organic etiology for their androgen deficiency; these benefits include increased muscle mass and strength, increased bone mineral density, improvement in sexual function and energy^45^. In addition, physiological testosterone replacement in young men was associated with a low frequency of side effects that include acne, oily skin, transient breast tenderness or gynecomastia, and erythrocytosis; these side effects were dose-dependent^45^. Therefore, testosterone regulation of renal fibrosis was further study in many aspects.

In conclusion, we have provided evidence supporting androgen as an anti-renal fibrosis medicine. Our studies provide the molecular basis for TP may alleviate the age-related renal fibrosis via suppression of TGF-β1/Smad signaling and activation of Nrf2-ARE signaling in aged rats.

## Materials and Methods

### Rats and TP injection

30 male Wistar rats were consisted of three groups (Experimental Animal Center of Hebei Medical University). The 6-month-old group (6Mon), aged vehicle control group (24Mon) and those with TP treatment group (24Mon-TP). 10 rats in each group. 24Mon-TP group rats received the 12 weeks (84-day) injection of subcutaneous TP (2 mg/kg/d at 5:00 PM to 6:00 PM) at the age of 21 months^16,46^. The 6Mon group rats and 24Mon group rats were received the sesame oil injection (vehicle). All rats were breed in a constant temperature room (22 ± 2 °C). All animal procedures were performed in accordance with the National Institutes of Health Guide for the Care and Use of Laboratory Animals, and were approved by the Local Animal Use Committee of Hebei Medical University.

### Histopathologic evaluation

The 5 rats of each group were anesthetized by intraperitoneal injection 4% chloral hydrate (300 mg/kg body weight) and perfused with saline transcardially. The rats received perfusion fixed by 4% paraformaldehyde in 0.1 M phosphate buffer (pH 7.4). Kidneys were separated and tissue fragments were post fixed in 4% paraformaldehyde in phosphate buffer for 4 h (4 °C). The renal tissue fragments of the rats were dehydrated in titrated ethanol, cleared in xylene and embedded in paraffin wax. Renal tissue section (5 μm) was gathered after sliced by a sliding microtome (Leica-RM2145, Germany) and then stained with H&E. The preparations were evaluated by light microscope and photographed (Olympus, BX 61, Japan).

### Masson’s trichrome

The renal tissue fragments which come from histopathologic evaluation were also used for Masson’s trichrome analysis. Interstitial fibrosis in the kidney was assessed from Masson’s trichrome-stained sections by modified Masson’s trichrome kits (Shijiazhuang JianFei biological technology co., LTD). Staining was quantified (40 objective) by image analysis. Randomly selected fields (20 per section) were digitized and subjected to color threshold analysis, (blue) giving a final average percentage positive stain per section.

### Transmission electron microscope (TEM)

The renal tissue fragments which come from histopathologic evaluation were also used for TEM analysis. The renal tissue fragments were post fixed for 2 *h* in the 3% paraformaldehyde and 1% glutaraldehyde in 0.1 M phosphate buffer (PB, pH 7.4). The fragments were fixed for 2 *h* using osmium tetroxide, dehydrated in ethanol, embedded for 48 *h* in araldite. The renal tissue was dissected and cut into sections (50 nm) by Leica UC-7 microtome. The sections were electronic staining by uranyl acetate (30 *min*) and lead citrate (10 *min*). The images were collected by transmission electron microscopy (Japan, Hitachi H-7500). Image-Pro Plus 6.0 image analysis software was used to measure the thickening of GBM and BBM. A total of 20 GBM and BBM were selected from each rat. The results presented the averaged thickening for each rat.

### Enzyme-linked immunosorbent assay (ELISA)

The five rats in each group were sacrificed by decapitation. Samples of trunk blood were collected and centrifuged at 4 °C. Serum samples were frozen at −80 °C until assessment. Serum BUN, Cre, UA, β2MG and CysC levels were detected by ELISA according to the manufacturer instructions. Rat ELISA kits obtained from Shanghai shuangying biological technology co., LTD.

### Oxidative stress parameters

The renal tissue was homogenized separately with 10 times (w/v) ice-cold 0.1 M PB (pH 7.4). The homogenates were used to assess oxidative stress parameters. MDA, LPO, GSH, GSH-px, CAT and SOD levels were measured spectrophotometrically using the detection kits of Nanjing Jiancheng Bioengineering Institute.

### Quantitative real-time polymerase chain reaction

Total RNA from the renal tissue obtained using Trizol reagent (Invitrogen, USA) following the instruction. RNA concentration was determined by measuring the absorbance (A) of a diluted sample at the 260 nm wavelength in a UV spectrometer. A total of 2 µg of total RNA was been subjected to reverse transcription to obtain cDNA template. The PCR was performed with 0.8 µl cDNA (diluted 1:10), specific primers 2 µl and the final volume of 20 µl. Initial cycle at 95 °C for 10 min, followed by 40 cycles. Then PCR products were analyzed. Expression of Collagen I, Collagen IV, fibronectin, MMP-2, MMP-9, TIMP-1, TIMP-2, TGF-β1, Smad 2, Smad 3, Smad 4, Nrf2, HO-1 as well as NQO1 genes were analyzed. The GAPDH was an internal control. The primers specific for the examined genes are shown in Table 1.

**Table 1:** Oligonucleotide primers sequences for mRNA amplification.

Primer	Direction	Sequence
Collagen I	Sense	ACTCAGCCGTCTGTGCCTCA
	Antisense	GGAGGCCTCGGTGGACATTA
Collagen IV	Sense	CCGGGATTTACTGGACCACC
	Antisense	CCCTTGCTCTCCCTTGTCA
Fibronectin	Sense	GACTCGCTTTGACTTCACCAC
	Antisense	TCCTTCCTCGCTCAGTTCGT
MMP-2	Sense	ACCTGGATGCCGTCGTGGAC
	Antisense	TGTGGCAGCACCAGGGCAGC
MMP-9	Sense	CGCTGGGCTTAGATCATTCC
	Antisense	TTGTCGGCGATAAGGAAGG
TIMP-1	Sense	GGGCTTCACCAAGACCTA
	Antisense	GAAGAAAGATGGGAGTGGG
TIMP-2	Sense	CCAAAGCGGTCAGTGAGA
	Antisense	TGGTGCCCGTTGATGTTC
TGF-β1	Sense	GACTCCTGCTGCTTTCTCC
	Antisense	GCGGTCCACCATTAGCAC
Smad 2	Sense	AACCCGAATGTGCACCATAAGAA
	Antisense	GCGAGTCTTTGATGGGTTTACGA
Smad 3	Sense	GTCAACAAGTGGTGGCGTGTG
	Antisense	GCAGCAAAGGCTTCTGGGATAA
Smad 4	Sense	AAGGCCTAGCACCACCTTAG
	Antisense	AGCCTTAAACTCTGACCTGT
Nrf2	Sense	GACCTAAAGCACAGCCAACACAT
	Antisense	CTCAATCGGCTTGAATGTTTGTC
HO-1	Sense	TGTCCCAGGATTTGTCCGAG
	Antisense	ACTGGGTTCTGCTTGTTTCGCT
NQO1	Sense	GGGGACATGAACGTCATTCTCT
	Antisense	AGTGGTGACTCCTCCCAGACAG
GAPDH	Sense	TGAACGGGAAGCTCACTG
	Antisense	GCTTCACCACCTTCTTGATG

### Western blot analysis

The renal tissues were homogenized in RIPA buffer. The homogenate was centrifuged (12,000 × *g*, 20 min, 4 °C), the supernatant was collected and centrifuged again. The Bradford method (Bio-Rad Laboratories, Hercules, CA) determined the protein concentration of the supernatant. Renal tissue (50 µg) protein samples were diluted with 2× sample buffer (50 mM Tris, pH 6.8, 2% SDS, 10% glycerol, 0.1% bromophenol blue, 5% b-mercaptoethanol) and heated for 5 min at 95 °C before SDS-PAGE on a 10% gel (Collagen I and Collagen IV were performed under native conditions, lack of this step), and subsequently transferred to PVDF membranes. Membranes were blocked with 5% skimmed milk 1 h at room temperature, and then were probed with mouse monoclonal anti-collagen I antibody (Abcam, ab90395, 1:1000), rabbit polyclonal anti-collagen IV antibody (Abcam, ab6586, 1:1000), rabbit monoclonal anti-fibronectin antibody (Abcam, ab45688, 1:5000), rabbit polyclonal anti-MMP-2 antibody (Abcam, ab37150, 1:200), rabbit polyclonal anti-MMP-9 antibody (Abcam, ab38898, 1:1000), rabbit polyclonal anti-TIMP-1 antibody (Abcam, ab61224, 1:500), rabbit polyclonal anti-TIMP-2 antibody (Abcam, ab180630, 1:500), goat polyclonal anti-TGF-β1 antibody (Santa-Cruz, C-16, sc-31609, 1:500), mouse monoclonal anti-Smad2/3 antibody (Santa-Cruz, A-3, sc-398844, 1:200), goat polyclonal anti-p-Smad2/3 antibody (Santa-Cruz, Ser 423/425, sc-11769, 1:200) and goat polyclonal anti-Smad 4 antibody (Santa-Cruz, C-20, sc-1909, 1:1000) overnight (4 °C). Membranes were washed three times using the phosphate buffered saline with 1% Tween 20, and then the IRDye® 800-conjugated goat anti-rabbit second antibody (1:10000, Rockland, Catalog: 611-145-002), rabbit anti-goat second antibody (1:10000, Rockland, Catalog: 605-445-002) or rabbit anti-mouse second antibody (1:10000, Rockland, Gilbertsville, Catalog: 610-445-002) incubated with the membranes for 1 h.

The renal tissue for the detection of Nrf2, HO-1 or NQO1 protein levels was tested based on our previous paper^46^. The membranes were incubated with polyclonal rabbit anti-Nrf2 antibody (Abcam, ab31163, 1:500), polyclonal rabbit anti-HO-1 antibody (Abcam, ab13243, 1:200), and mouse anti-NQO1 monoclonal antibody (Abcam, ab28947, 1:200) overnight (4 °C). IRDye® 800-conjugated goat anti-rabbit second antibody (1:3000, Rockland, Catalog: 611-145-002) or goat anti-mouse second antibody (1:3000, Rockland, Catalog: 610-145-002) was incubated with the membranes for 1 h.

Odyssey infrared scanner (LI-COR Biosciences) analyzed the relative density of bands. The densitometry values were normalized with respect to the values of anti-histone 3 (H3, 1:1000, bioWORLD,) for Nrf2 and anti-β-actin (1:3000, Santa Cruz) for Collagen I, Collagen IV, fibronectin, MMP-2, MMP-9, TIMP-1, TIMP-2, TGF-β1, Smad2/3, p-Smad2/3, HO-1 as well as NQO1 immunoreactivity.

### Immunohistochemistry and densitometric analysis

The renal tissue fragments which come from histopathologic evaluation were also used for immunohistochemical analysis. Immunohistochemical staining was based on conventional methods. All sections were subjected to deparaffinized, hydrated, antigen repaired (0.01 M citrate buffer, pH 6.0) and goat serum closed. The sections received an overnight incubation with mouse anti-NQO1 monoclonal antibody (Abcam; ab28947, 1:500) at 4 °C. The sections were incubated with biotinylated goat anti-mouse IgG (2 h, Jackson ImmunoResearch; Code 115-065-003, 1:300) and horseradish peroxidase-conjugated streptavidin (1 h, 1:300) successively at room temperature. All sections were stained for 5 min in 0.05 M Tris-HCl buffer (containing 0.05% Diaminobenzidine and 0.03% H_2_O_2_, pH 7.6). The average optical density (AOD) of NQO1 immunoreactive intensity was measured. Ten sections were measured in each rat. The averaged AOD value of NQO1 was presented for each rat.

### Serum testosterone level assay

The trunk blood of 5 rats was collected in each group. The serum was obtained by centrifugation (3,000 × *g*, 20 min). Serum testosterone concentration was detected by radioimmunoassay according to the protocol of the kit. The testosterone radioimmunoassay kit was purchased by the Tianjin Nine Tripods Medical and Bioengineering Co., Ltd. China.

### Statistical analyses

All data were presented as the mean ± SD. The normality and homogeneity variance were tested by Kolmogorov–Smirnov test and Levene’s test respectively. The data conformed both normal distribution (*P* å  0.1) and homogeneity of variance (*P* å  0.1), one-way analysis of variance (one-way ANOVA) followed by a Student–Newman–Keuls (SNK) for multiple comparisons was performed. Otherwise, the non-parametric statistics (Kruskal–Wallis test) followed by a Mann–Whitney U between groups were done. The *P* < 0.05 was considered statistically significant.

## Electronic supplementary material

Supplementary information
