# Supplementary material for: Amelioratory Effects of Testosterone Propionate on Age-related Renal Fibrosis via Suppression of TGF-β1/Smad Signaling and Activation of Nrf2-ARE Signaling
Source: Sci Rep. 2018 Jul 16;8:10726. doi: 10.1038/s41598-018-29023-3 (PMC6048025; doi:10.1038/s41598-018-29023-3)
Supplement: Supplementary file 1 — Supplementary information [file 41598_2018_29023_MOESM1_ESM.pdf]

Amelioratory Effects of Testosterone Propionate on Age-related Renal Fibrosis via Suppression of TGF-β1/Smad Signaling and Activation of Nrf2-ARE Signaling

Guoliang Zhang[1, 2], Yunxiao Kang[1], Chenming Zhou[3], Rui Cui[2], Min Jia[4], Shen Hu[5], Xiaoming Ji[1], Jiayu Yuan[1], Huixian Cui[3,6], Geming Shi[1,6]

[1] Department of Neurobiology, Hebei Medical University, Shijiazhuang, 050017, PR China; [2] Department of Human Anatomy, Hebei Medical University, Shijiazhuang, 050017, PR China; [3] Department of electron microscopy center, Hebei Medical University, Shijiazhuang, 050017, PR China; [4] Department of urology, Hebei Civil Affairs General Hospital, Xingtai, 054000, PR China; [5] Department of urology, Handan Second hospital, Handan, 056001, PR China. [6] Neuroscience Research Center, Hebei Medical University, Shijiazhuang 050017, PR China.

Guoliang Zhang and Yunxiao Kang contributed equally to this work. Correspondence and requests for materials should be addressed to G. S. (email: shigeming@163.com).

Supplementary Information

S1**:** Orignial Western image for Figure 3.

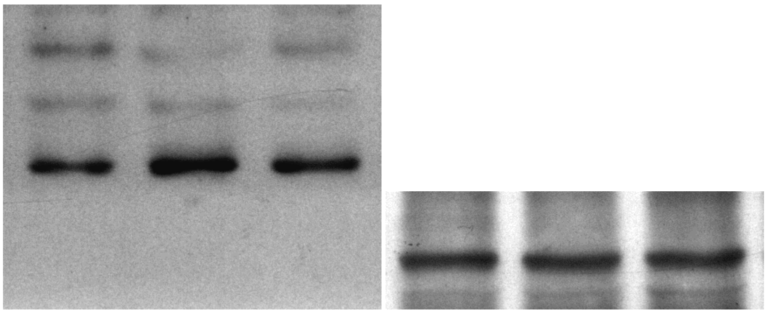

Collagen I β-actin

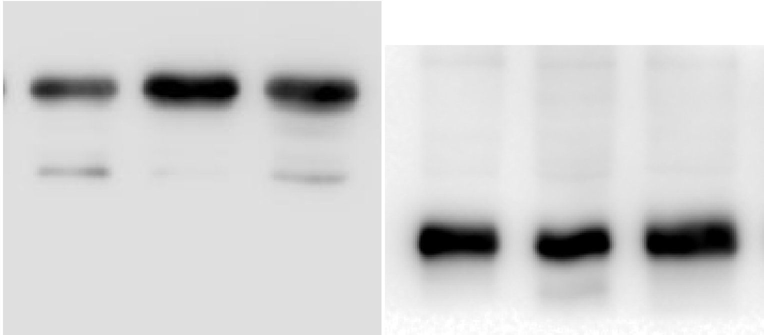

Collagen IV β-actin

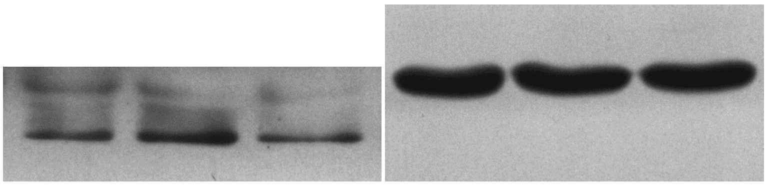

fibronectin β-actin

S2**:** Orignial Western image for Figure 4e.

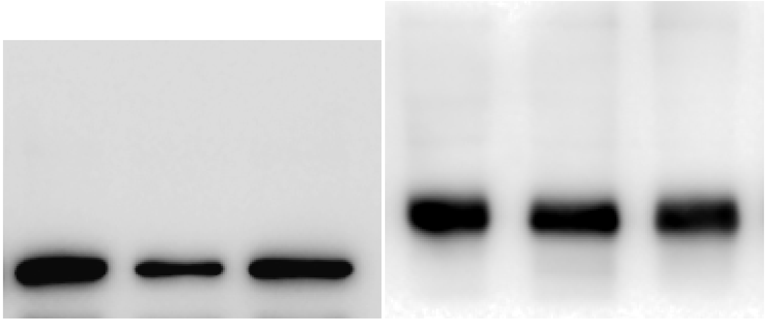

MMP-2 β-actin

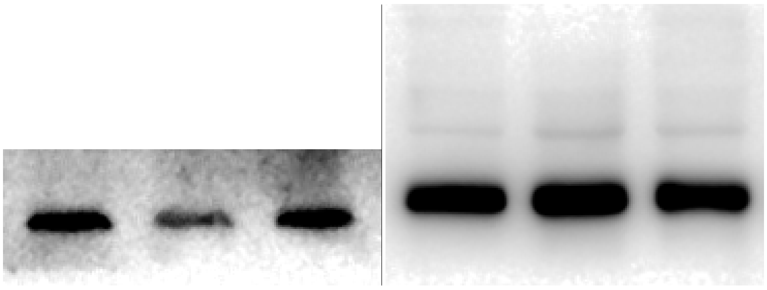

MMP-9 β-actin

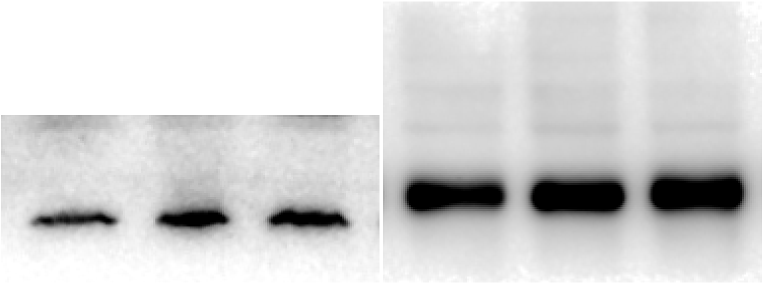

TIMP-1 β-actin

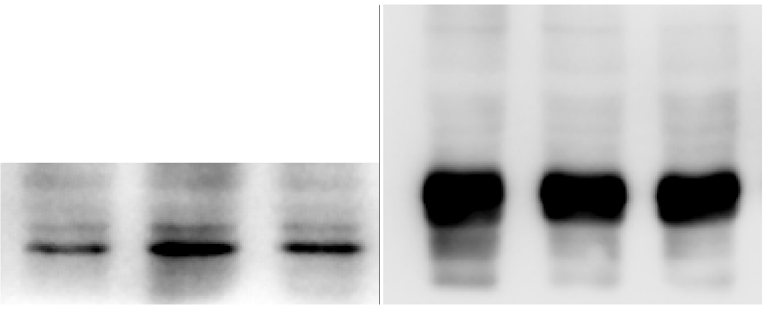

TIMP-9 β-actin

S3**:** Orignial Western image for Figure 5e.

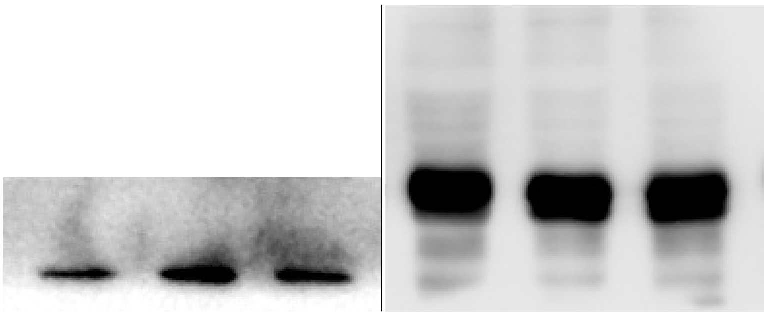

TGF-β1 β-actin

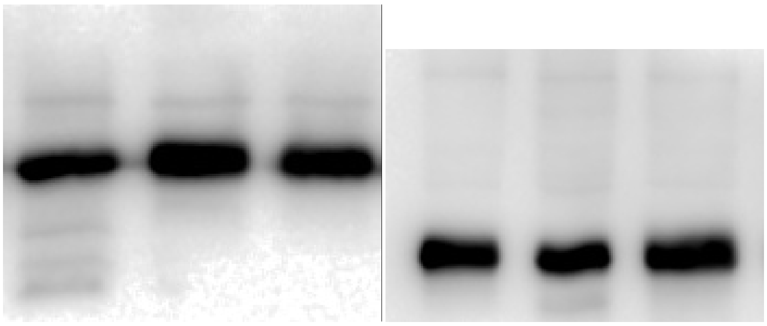

Smad2/3 β-actin

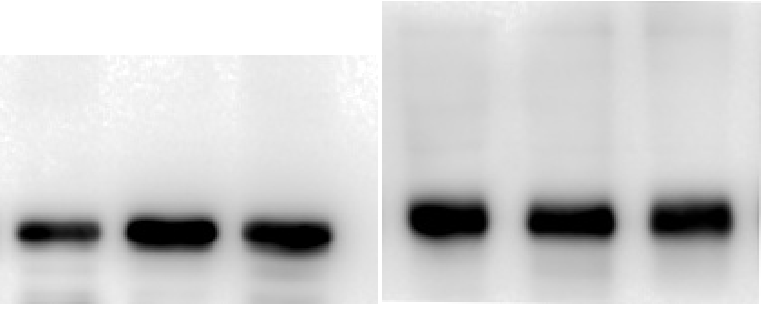

p-Smad2/3 β-actin

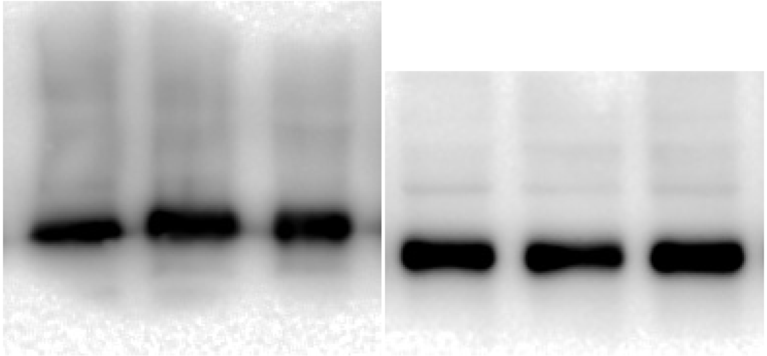

Smad4 β-actin

S4**:** Orignial Western image for Figure 7d.

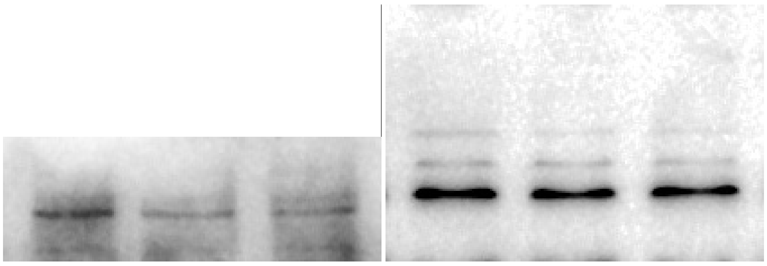

Nrf2 H3

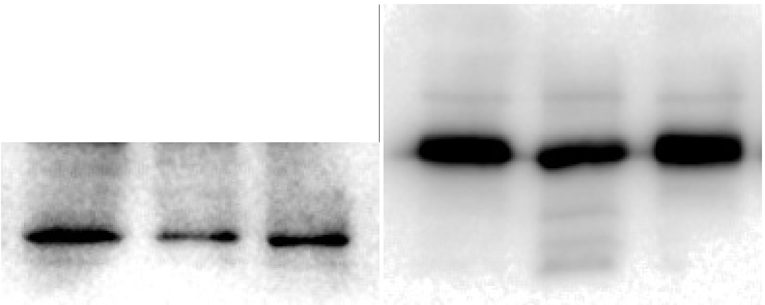

HO-1 β-actin

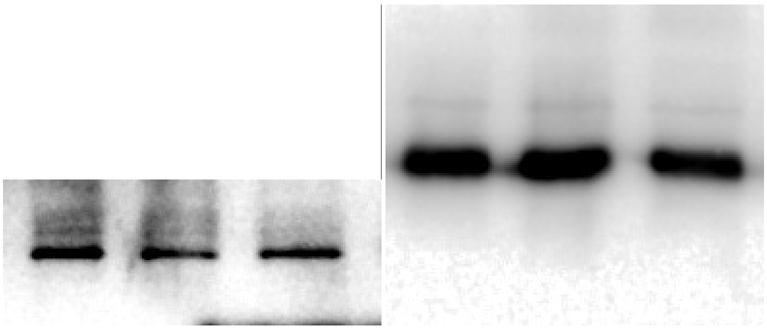

NQO1 β-actin
